# Spontaneous Fracture of the Femora

**Published:** 1909-11-06

**Authors:** D. M. Macdonald


### The General Practitioner's Column.

[Contributions to this Column are invited, and, if accepted, will be paid for.]

### SPONTANEOUS FRACTURE OF THE FEMORA.

By D. M. MACDONALD, M.D.

A curious illustration of the above, not without some interesting features, seems worthy of recording. A boy 17 years of age, imbecile but fairly well-developed, who had never talked or walked, was in the habit of sleeping with his left big toe in his mouth, a condition rendered possible by the extreme laxity of all the joints of the body. On the morning of May 28 a large swelling was discovered over the left thigh, and it was also noted that he was unable to draw up the leg, but otherwise there was not much apparent inconvenience and the fracture remained undetected by the parents. Three days afterwards he was medically attended and found to have fractured the left femur in its lower third. It was put up in the double inclined position, and subsequently healed. A restraining band was put on to prevent him raising the foot to the head in the future. On June 11 an accident caused in a similar fashion befell the upper third of the right femur. The line of cleavage was sharp and the fracture almost compound. It was put up by the extension method, but absolutely refused to unite. Ultimately, through degenerative changes, a wound was produced with extensive sloughing of the parts, and the limb was amputated. From the condition of the patient an antecedent pathological change in the bones was expected, but the subsequent examination of the bone only evidenced faulty union, there being an attempt at bone formation. The rest of the bone apart from the fracture was hard and quite normal.

